# Pharmacological prevention of postictal agitation after electroconvulsive therapy—A systematic review and meta-analysis

**DOI:** 10.3389/fpsyt.2023.1170931

**Published:** 2023-04-20

**Authors:** Thomas C. Feenstra, Yvonne Blake, Adriaan W. Hoogendoorn, Krista Koekenbier, Aartjan T. F. Beekman, Didi Rhebergen

**Affiliations:** ^1^GGZ Centraal Mental Health Care, Amersfoort, Netherlands; ^2^Mental Health Program, Amsterdam Public Health Research Institute, Amsterdam, Netherlands; ^3^Department of Psychiatry, Amsterdam UMC Location Vrije Universiteit Amsterdam, Amsterdam, Netherlands; ^4^GGZ InGeest Mental Health Care, Amsterdam, Netherlands; ^5^Department of Psychiatry, Leiden University Medical Center, Leiden, Netherlands

**Keywords:** electroconvulsive therapy, ECT, cognitive side effects, postictal agitation, dexmedetomidine

## Abstract

**Background:**

Postictal agitation (PIA) after electroconvulsive therapy (ECT) is a serious clinical problem estimated to occur in 7–36% of patients and recur in 19–54% of patients. PIA has the potential to cause dangerous situations for the patient and staff members aside from the financial impact. To date, it is unclear which pharmacological interventions should be used in the management of PIA. This study aimed to systematically review the (preventative) pharmacological treatment options for PIA after ECT.

**Method:**

A systematic search was done in PubMed, EMBASE, PsycINFO, and Web of Science from inception until 10 November 2022. We included randomized trials with any pharmacological intervention or comparison and a predefined outcome measure on PIA. Studies that solely included patients with neurodegenerative disorders or stroke were excluded. Data quality was assessed with the RoB2 and GRADE. Meta-analysis was performed if possible. This study was registered on PROSPERO under CRD42021262323.

**Results:**

We screened 2,204 articles and included 14 studies. Dexmedetomidine was investigated in 10 studies. Alfentanil, lignocaine, esmolol, midazolam, propofol, ketamine, haloperidol, and diazepam were each studied in only one study. Meta-analysis revealed an OR of 0.45 (0.32–0.63), a moderate effect size, in favor of dexmedetomidine than placebo to prevent PIA with very low heterogeneity (I^2^ = 0%). The certainty of the evidence was moderate. The other interventions studied were all found to have low certainty of evidence.

**Conclusion:**

For clinical practice, we believe that our results indicate that dexmedetomidine should be considered for the prevention of PIA in patients that have previously experienced PIA.

## 1. Introduction

Electroconvulsive therapy (ECT) is an important treatment modality in psychiatry. The effectiveness of electroconvulsive therapy is particularly evident in (treatment-resistant) severe depression, where it is seen as the most effective biological treatment (1). Despite the effectiveness of ECT, its use around the world is less than may be expected (2). While limited availability and stigma are the main contributors to this discrepancy, cognitive side effects are an important consideration for clinicians when prescribing ECT (3, 4).

Postictal agitation (PIA), or emergence agitation, is one of the multiple clinical syndromes that fits within the ECT-related cognitive side effects. It occurs upon awakening after ECT and is seen in approximately 7–36% of patients (5–9). There is a significant clinical heterogeneity in the severity of PIA, but in a significant amount of cases, PIA causes dangerous situations. These dangerous situations can cause trauma in patients or staff members, financial losses due to damaged equipment, and inefficiency in the lead times (5, 10). Furthermore, patients who have experienced PIA are at an increased risk of recurrence, estimated at 19–67% (5–9).

In cases where pharmacological treatment for PIA is needed, it is frequently too late to avoid the abovementioned adverse effects. In addition, it has been demonstrated that first-line treatment with benzodiazepines has proven ineffective once PIA has occurred (11). Preventative (pharmacological) treatment might be a solution to this problem. Although previous research is sparse on this topic, dexmedetomidine, a selective alpha-2 receptor agonist, has gained traction over the years as a possible drug to mediate hyperdynamic responses to ECT (i.e., hypertension, tachycardia, and postictal agitation). Thus far, the literature on dexmedetomidine shows inconclusive results (12–15). Studies on other pharmacological interventions (e.g., benzodiazepines and antipsychotic medication) are even more sparse. To the best of our knowledge, the published literature on the treatment of PIA has not resulted in an international consensus document on its management. This systematic review and meta-analysis aimed to summarize the current evidence of pharmacological (preventative) interventions for PIA after ECT.

## 2. Methods

### 2.1. Protocol and registration

This review follows the PRISMA guidelines for reporting systematic reviews (16). The protocol was pre-registered on the PROSPERO registry with ID CRD42021262323 on 9 November 2021. Ethics approval was not required for this review as no new data were collected.

### 2.2. Search strategy

A comprehensive systematic search was done in the bibliographic databases PubMed, Embase, PsycINFO (EBSCO), and Web of Science (Clarivate) from inception to 10 November 2022. The search strategy was developed in collaboration with a medical information specialist. Search terms included controlled terms from MeSH in PubMed and EMtree in EMBASE.com, thesaurus terms in PsycINFO as well as free text terms related to ECT (i.e., electroconvulsive therapy, electroconvulsive treatment, and shock therapy) and confusion (i.e., agitation, excitement, restlessness, delirium, disorientation, and hyperdynamic state). Details of the search strategy are available as supplementary material (Supplementary Table 1).

### 2.3. Inclusion and exclusion criteria

We included all studies that were randomized trials of adult patients undergoing electroconvulsive therapy for any psychiatric indication; compared any periprocedural pharmacological interventions and any comparison (i.e., no medication, placebo, and other medications); had a standardized predefined outcome scale on postictal agitation [i.e., Richmond Agitation Sedation Scale (RASS), emergence agitation score, or any other Likert-type scale]; and were written in the English language.

We excluded studies that solely included patients with neurodegenerative disorders or a history of stroke and that only compared differences in electroconvulsive therapy characteristics.

### 2.4. Screening and data extraction

**Figure 1:**
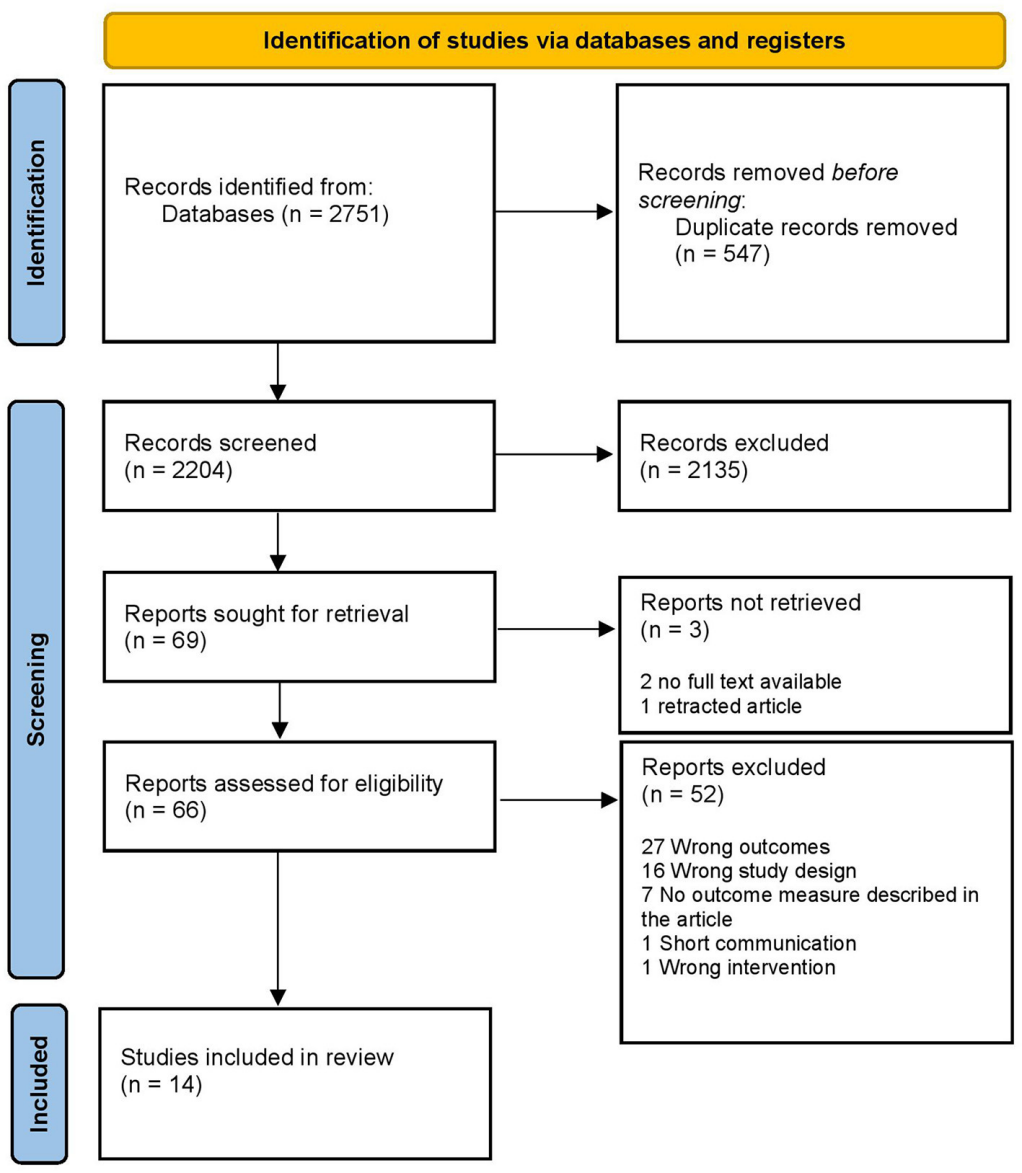
Flow diagram of the search process.

The search results were independently screened by three reviewers (TCF and KK screened before 18 June 2019 and TCF and YB from 18 June 2019 to 10 November 2022). Duplicates were removed using Endnote up to 18 June 2019 (after a check from the reviewer) and through Covidence systematic review software for Windows (Veritas Health Innovation, Melbourne, Australia; available at www.covidence.org) from 18 June 2019 to 10 November 2022. The full-text review was done by three reviewers (TCF and KK before 18 June 2019, TCF and YB from 18 June 2019 to 10 November 2022). The flow diagram shows the results of the search process (Figure 1). If the full-text article was unavailable or unclear, the corresponding author was contacted via email. Data extraction was designed and subsequently executed and double-checked by the first author (TCF) and checked by the second author (YB). In case of disagreement in any of the steps (i.e., screening, full-text review, risk of bias assessment, and data extraction), a third reviewer was consulted (DR).

### 2.5. Outcome measure

We extracted the data on PIA as a dichotomous outcome (i.e., PIA or no PIA), as opposed to the use of continuous outcome scales (e.g., emergence agitation scale and RASS), to improve the clinical relevance of the outcome for our research question, and to harmonize the different outcome scales. When PIA was not predefined in the available (continuous) outcome scores, we set a cutoff based on the score that described PIA the best (e.g., “restlessness” or “aggression requiring a nurse at the bed continuously”). The reason to dichotomize the PIA outcome measure can be illustrated briefly by the emergence agitation scale (EAS) (17). The EAS is a Likert-type scale from 1 to 5, where PIA can be defined as a score of 3 (irritable and noisy) or higher (15, 18). Scores below 3 indicate sleeping [1/5] or awake and relaxed patients [2/5]. If, for example, a study compared two groups with a mean emergence agitation score of 1.4 and 2, these means would not accurately reflect the difference in PIA occurrence in that study. In this example, a dichotomous descriptive statistic (PIA vs. no PIA) or odds ratio (OR) for PIA cases answers our research question more accurately and was therefore reported.

### 2.6. Risk of bias assessment/study quality

Two authors (TCF and YB) independently reviewed the full-text articles based on their methodological quality, using the revised Cochrane Collaboration Risk of Bias tool (RoB 2.0) (19). For cross-over trials, we used the variant of the RoB 2.0 for cross-over trials developed by the same group.

### 2.7. Meta-analysis

We conducted a meta-analysis for each pharmacological intervention that was investigated in more than one trial using the metan command in Stata 17.0 software for Windows. Due to expected heterogeneity in population and effect, a random-effects model was used (20). To synthesize an optimal amount of data per pharmacological intervention, we decided to bundle dose differences (i.e., dexmedetomidine 0.2, 0.5, and 1 mcg/kg) in the same meta-analysis.

Odds ratios (ORs) and their 95% confidence interval (CI) were computed for the included studies based on the individual patient data if this was not already done in the original manuscript. For cross-over trials, the Mantel–Haenszel OR for paired outcomes is calculated as the ratio of discordant pairs. For the exact 95% CI, the standard error was estimated from the width of the 95% CI by division by 2 × 1.96. When the OR could not be calculated as a result of division by zero, a fixed value of 0.5 was added to all the cells, which is a common method if the arm sizes are balanced (21). To correct the (standard error of the) OR for the repeated observations in parallel studies with multiple ECT sessions per patient, an intra-class correlation (ICC) was used. As original patient data on PIA prevalence and recurrence from the included studies were not available, and consensus on PIA prevalence and recurrence in the international literature was absent, we approximated the ICC through a small simulation study based on the mean PIA prevalence (21,5%) and PIA recurrence (43%) from five previous studies using seven repeated measurements (5–9). The ICC was then used to adjust the standard error of the lnORs. lnOR from cross-over trials and parallel trials was merged in the final meta-analysis (22). Heterogeneity was measured using the Q test and reported using the I^2^ statistic. I^2^ quantifies the percentage of total variation across studies considered to be due to heterogeneity rather than chance. I^2^ values of 25, 50, and 75% indicate low, moderate, and high heterogeneity, respectively (23).

### 2.8. Sensitivity analysis

Sensitivity analyses were done for the lower-end [7% prevalence (9) and 19% recurrence (7)] and upper-end extremes [36% prevalence (8) and 67% recurrence (5)] of PIA prevalence and recurrence estimates to assess the robustness of the synthesized results.

### 2.9. Publication bias

The possibility of publication bias was assessed through the creation of a funnel plot. The interpretation was done with the eye-balling method or, if >10 studies were included in the meta-analysis, through the Egger test (24).

### 2.10. Certainty of evidence

We used the GRADE approach to determine the certainty of the evidence and justified all downgrade and upgrade decisions using footnotes (25). We created a summary of findings table with the sum of available data on the main outcome, including the magnitude and direction of the effect of all interventions, with the GRADEpro Guideline Development Tool software for Windows (McMaster University and Evidence Prime, 2022; available from gradepro.org).

## 3. Results

### 3.1. Search results

In total, 2,204 studies were screened, of which 66 studies were eligible for full-text review. After review, we included 14 studies (13–15, 17, 18, 26–34). Dexmedetomidine was investigated in 10 studies. The other interventions (i.e., alfentanil, lignocaine, esmolol, midazolam, propofol, ketamine, haloperidol, and diazepam) were all studied in one study. The duplicates, reasons for exclusion, and extracted studies are reported in Figure 1.

### 3.2. Patient characteristics

The reviewed studies included a total of 735 unique patients studied in 3,595 ECT sessions. The average age of the patients was 34 years old, and 58% were women. The specific psychiatric indication for ECT was specified in only three of the 14 studies (i.e., depression). One study (17) specifically included patients that had experienced PIA before. All the included studies and patient characteristics are presented in Supplementary Table 2.

### 3.3. PIA prevalence and recurrence

Based on the five previous studies, with a median PIA prevalence of 21.5% and median PIA recurrence of 43.0%, we computed an ICC of 0.428. Sensitivity analyses were done on the lower-end ICC of 0.196 and upper-end ICC of 0.697.

### 3.4. Dexmedetomidine

Dexmedetomidine was the most frequently investigated intervention drug in five parallel (13, 18, 29–31, 35) and five cross-over designs (14, 17, 26–28). It was studied in 617 patients during 1,537 sessions and exclusively utilized as a preventative intervention (i.e., dexmedetomidine was given before the anesthetic was given). The specific psychiatric indication for ECT was specified in only one of the studies (i.e., depression). In eight out of 10 studies, a dose of 0.5 mcg/kg was studied, 1 mcg/kg was studied in three studies, and 0.2 mcg/kg was studied in one large study (18). All studies had some concerns as to the risk of bias or a high risk of bias. The risk of bias assessment for all included studies is found in the supplementary material (Supplementary Figure 1).

**Figure 2:**
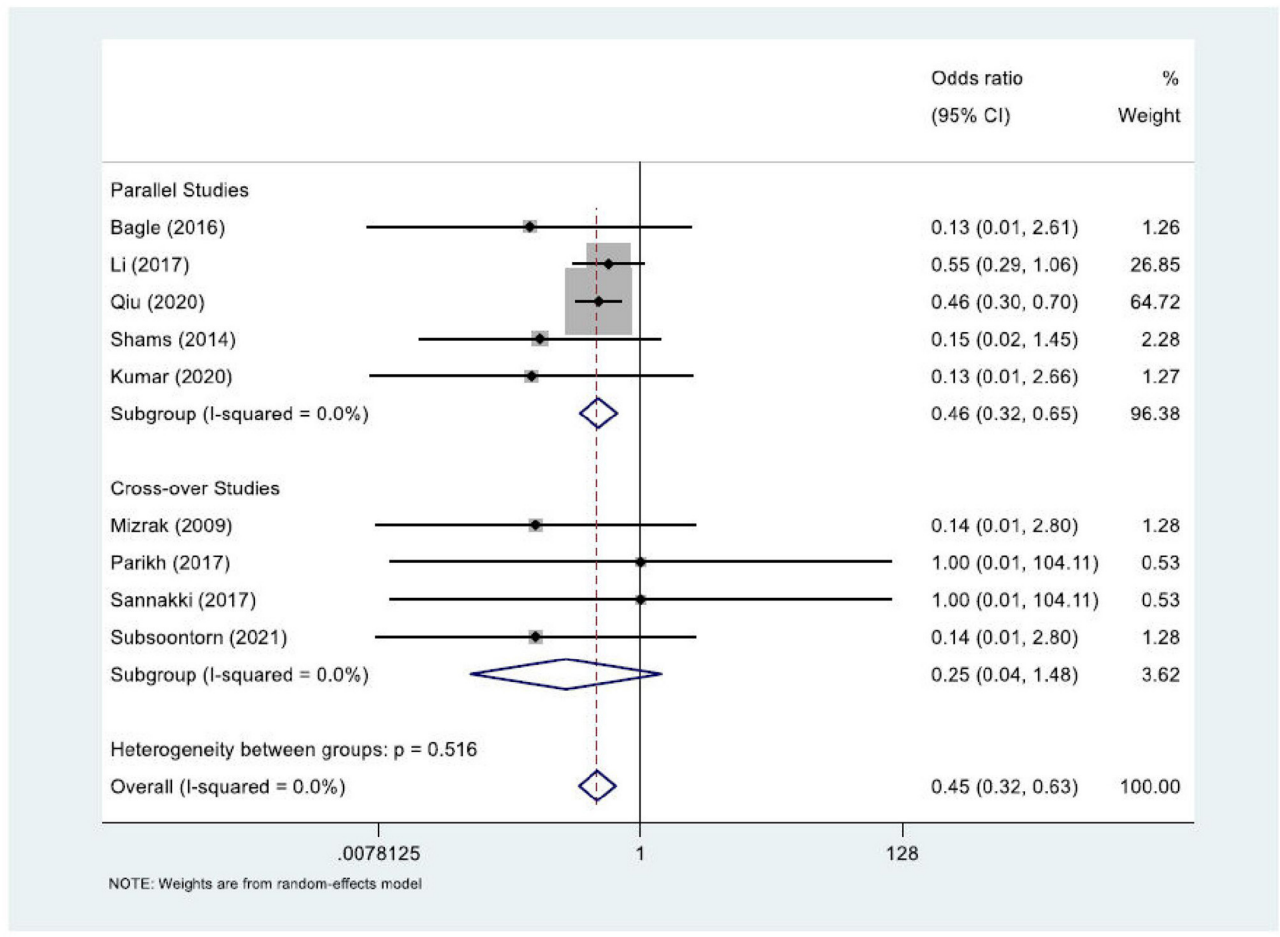
Forest plot of all studies that investigated dexmedetomidine (vs, placebo) in the treatment of PIA.

**Figure 3:**
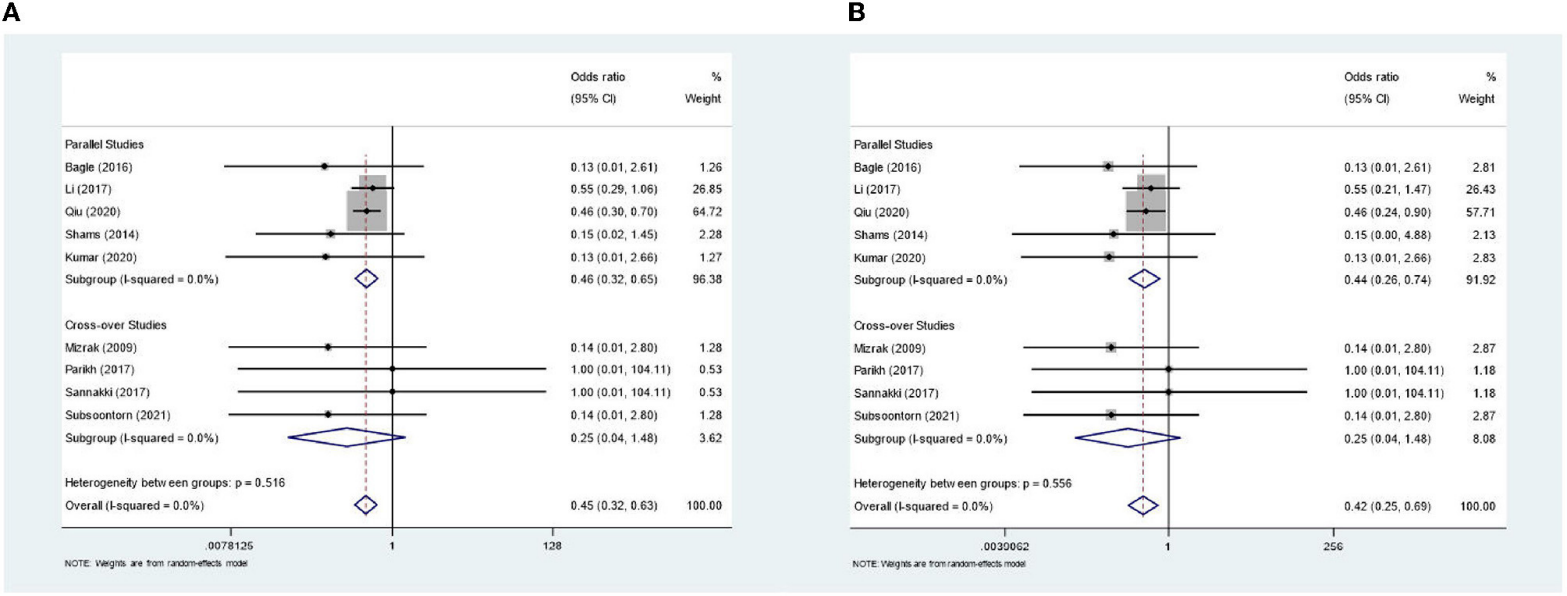
Sensitivity analyses for lower- and upper end intra-class correlation. **(A)** Forest plot for lower-end value of intra-class correlation (=0.196). **(B)** Forest plot for upper-end value of intra-class correlation (=0.697).

**Figure 4:**
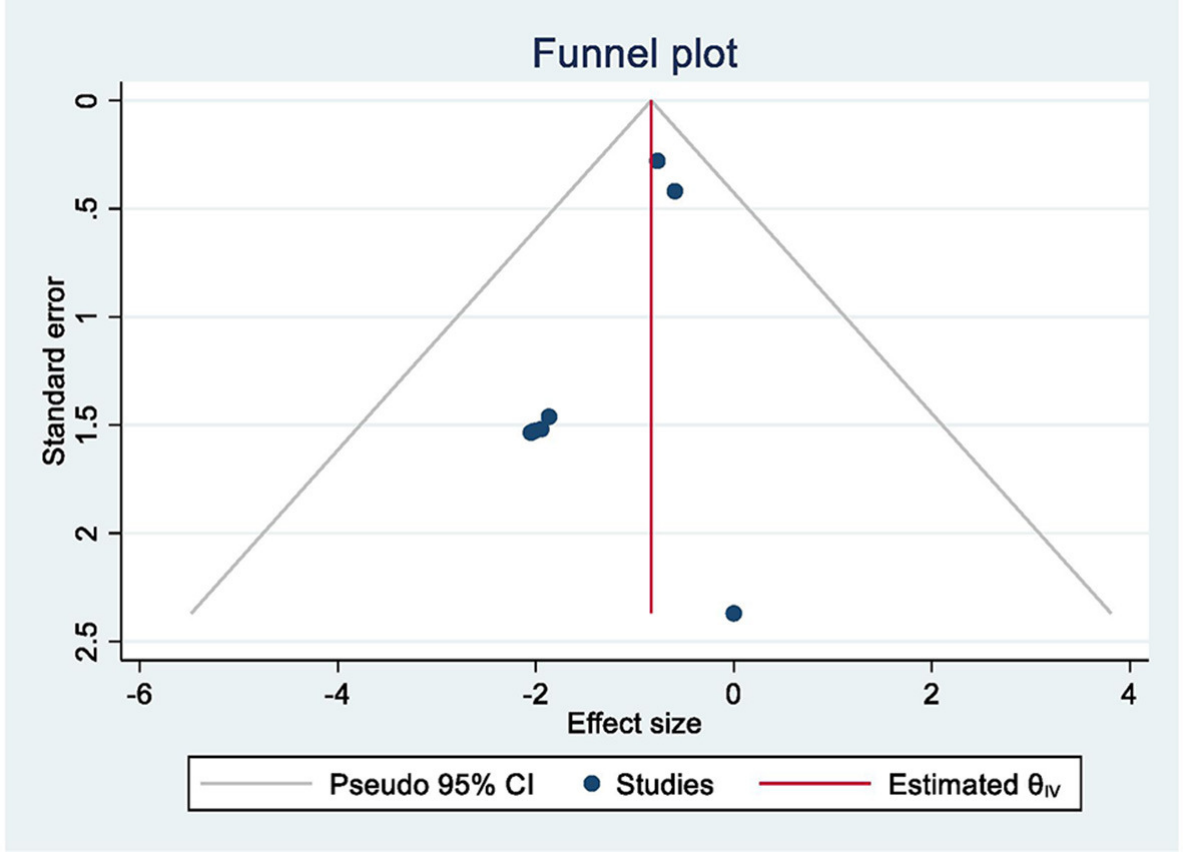
Funnel plot of all studies that investigated dexmedetomidine (vs. placebo).

In the meta-analysis, shown in Figure 2, we included 583 patients from nine out of 10 studies. One study was not usable (26) as it only reported on mean agitation scores in groups; therefore, the amount of PIA cases was not clear. Two studies (13, 18) comprised >80% of the weight. The combined odds ratio for dexmedetomidine was 0.45 (0.32–0.63), which corresponds to Cohen's *d* of 0.44 and a moderate effect size. Cochran's Q test for heterogeneity showed low heterogeneity with an I^2^ of 0.0%. The certainty of the evidence was considered moderate. The sensitivity analyses for an ICC of 0.196 and 0.697 showed an OR of 0.45 (0.32–0.63) and 0.42 (0.25–0.69), respectively, indicating a robust result (Figure 3). Publication bias was investigated with a funnel plot (Figure 4). The funnel plot showed relative symmetry, despite few included studies, possibly indicating that no clear publication bias is present. The Egger test was not used as only nine studies were included in the meta-analysis. The summary of evidence tables, according to GRADE, for dexmedetomidine and the other interventions is shown in Supplementary Table 3.

### 3.5. Other drugs

#### 3.5.1. Alfentanil

Alfentanil was studied in 25 patients in a cross-over design and compared with dexmedetomidine and placebo (26). They found no significant difference between alfentanil and placebo, with mean emergence agitation scores of 1.84 (0.37) and 2.06 (0.04), respectively. The comparison between dexmedetomidine and placebo is included in the meta-analysis. Individual patient data on PIA prevalence (defined as an emergence agitation score of 3 or higher) and recurrence were not available. The risk of bias in this study was considered high, and the certainty of the evidence that alfentanil does, or does not, have an effect on PIA prevalence was considered low.

#### 3.5.2. Esmolol and lignocaine

Parikh et al. (27) studied the effects of esmolol, a selective beta-adrenergic antagonist, and lignocaine, along with two doses of dexmedetomidine and a placebo condition in a five-way cross-over double-blind randomized trial on 30 patients in 150 sessions. PIA was not seen in any of the sessions. The risk of bias in this study was considered to be intermediate (some concerns), and the certainty of the evidence that esmolol or lignocaine could prevent or not prevent PIA was considered to be low.

#### 3.5.3. Midazolam

Mizrak et al. (17) studied midazolam 0.025 mg/kg given as a preventive intervention (i.e., pre-ECT) in a three-way cross-over design in 15 patients with dexmedetomidine and a placebo condition. They found a statistically significant difference in mean emergence agitation scores in favor of midazolam when compared to placebo. These mean scores were reported in a graph and were unextractable. Furthermore, they reported that emergence agitation scores of 4 and 5, situations where they gave additional drugs to control the agitation, were present in three patients in the control group and zero patients in the midazolam group. The authors performed no statistical analysis on this difference. The risk of bias was considered to be intermediate, and the certainty of the evidence that midazolam given before ECT could prevent PIA was considered to be low.

#### 3.5.4. Propofol and ketamine/propofol

Propofol was studied as the sole anesthetic and as an addition to anesthesia directly after ECT. Gaddam et al. (15) studied propofol as the sole anesthetic in a cross-over design in 30 patients, with ketamine/propofol as another intervention and thiopentone as the comparison. They found a statistically significant difference in mean agitation scores in favor of propofol and ketamine/propofol when compared with thiopentone. They also reported agitation scores >2, indicating PIA, in 3/30 sessions in the ketofol group and 0/30 sessions in the propofol group, compared to 7/30 sessions in the thiopentone group. Statistical analysis was not performed on these results. The risk of bias in this study was considered high, and the certainty of evidence that there is any preventative effect of these interventions was considered low.

Propofol as an addition after anesthesia (with etomidate) was studied by Tzabazis et al. (32) in a cross-over design with 13 patients and 74 sessions compared to no intervention. They reported a statistically significant reduction of the PIA prevalence in the propofol group (3/37 sessions), compared to the control group (8/37 sessions). The risk of bias was considered high, and the certainty of the evidence that propofol after ECT can prevent PIA was considered low.

#### 3.5.5. Ketamine

Rasmussen et al. (33) compared ketamine to methohexital as the main anesthetic in a parallel design in 35 patients (166 sessions) with unipolar or bipolar depression without a primary psychotic disorder. They found no statistically significant difference in the presence of postictal agitation between groups when comparing mean agitation scores. The risk of bias was considered high, and the certainty of the evidence that ketamine does not prevent PIA was considered low.

#### 3.5.6. Haloperidol and diazepam

Gomez et al. (34) studied haloperidol 20 mg and diazepam 20 mg pre-ECT in a cross-over design in comparison with no medication. They reported a statistically significant difference in mean agitation scores, 0.27 in the haloperidol group, 0.36 in the diazepam group, and 1.25 in the control group. As agitation scores of 2 and higher in this study indicate PIA and no data on patients with scores of 2 or higher were reported, there are insufficient data to compare the interventions. The risk of bias in this study was considered high, and the certainty of the evidence that haloperidol or diazepam could work as prevention for PIA was considered low.

## 4. Discussion

### 4.1. Summary of main findings

This systematic review is the first, to the best of our knowledge, to comprehensively study the evidence for pharmacological interventions to prevent postictal agitation (PIA) after ECT. We found that dexmedetomidine, lignocaine, esmolol, midazolam, propofol, ketamine, haloperidol, and diazepam were previously investigated in randomized trials for their effectiveness in preventing PIA after ECT. Dexmedetomidine was the only intervention on which meta-analysis was possible. The meta-analysis (*k* = 9) showed an OR of 0.45 (0.32–0.63) in favor of dexmedetomidine, which was given preventively (i.e., before anesthesia) in all trials in doses of 0.2–1 mcg/kg. Based on this systematic review, meta-analysis, and GRADE approach, there is moderate certainty of evidence that dexmedetomidine prevents PIA in ECT more than placebo. All other drugs were tested in only one study each, with varying comparators and on small numbers of patients. We found a low certainty of evidence that any of the other (preventive) pharmacological interventions reduce the risk of PIA in ECT.

### 4.2. Comparison with existing literature

The use of dexmedetomidine to prevent PIA after ECT was the most recently described in two reviews that summarized the evidence thus far (12, 36). In contrast to our results, Li et al. (12) performed a meta-analysis on two studies (17, 26) to investigate the effect of dexmedetomidine on PIA and found no statistically significant preventative effect. The newly published studies, since this meta-analysis, largely explain the difference with our findings. In addition, in their meta-analysis, they decided to analyze the difference in mean EAS scores and therefore included a study that we excluded from the meta-analysis (26) (as we could not acquire the amount of PIA cases in the groups). As stated in the Method section, for our research question, we believe that PIA, as a clinical entity, is best represented in a dichotomous analysis (i.e., PIA vs. no PIA). The mean EAS scores for the intervention or control group in the studies that comprised this meta-analysis did not reach a score of 3 (where PIA equals a score of 3 or higher). Therefore, the effect of dexmedetomidine (to reduce the mean EAS score as opposed to placebo) in this meta-analysis was mostly measured on “relaxed and awake” patients. In addition, Sterina et al. (36) recently published a narrative review on the acute and prophylactic interventions for PIA after ECT. They described various (non-) pharmacological interventions that could be attempted to prevent or treat PIA, including dexmedetomidine. Promethazine, melatonin, and antipsychotics (e.g., olanzapine) were suggested interventions that were not included as none of the mentioned studies met the inclusion.

### 4.3. Strengths and limitations

The main strengths of this study are strict inclusion and exclusion criteria and the utilization of the ICC to correct repeated observations in the meta-analysis. This allowed us to produce robust results and improve the clinical applicability of the research findings in this understudied field. While these strengths could make this study important for clinicians facing (recurrent) PIA in the future, there are several limitations that should be considered in the interpretation of our results. These limitations include [1] the poorly defined research population, [2] limited (geographical) generalizability, [3] the unvalidated outcome score, [4] clinical diversity under the PIA phenotype, and [5] the overall high risk of bias in the included studies. As seen in Supplementary Table 2, none of the included studies clearly defined the psychiatric indication for ECT, management of agitation prior to ECT or in between sessions, the ECT procedure, or any other possible risk factors for PIA that could confound the results. These risk factors include gender (6, 35), concomitant lithium or SSRI use (6, 36), and substance use disorder (35). The generalizability of the results is questionable as most studies were performed in Asia [except for one Egyptian study (29)], and mostly young healthy patients or patients with mild systemic disease (ASA II) were included. The generalizability and applicability to clinical practice are further hampered by unknown inter-rater reliability of the outcome measures (except in the case of the Richmond agitation-sedation scale) and the heterogeneous nature of PIA. PIA remains a phenotypical description of the possibly diverse underlying pathophysiology. Qiu et al. (13) made an important step in unraveling PIA, by defining postictal delirium (PID) and PIA separately. This subtyping of patients experiencing PIA is needed to reduce confounding and improve generalizability.

With regard to our meta-analytic techniques, the conversion to a dichotomous outcome of PIA instead of study-specific continuous agitation scores, and the correction with the ICC, caused a change in the statistical significance of the outcome in four included studies. This effect was seen in both ways. Qiu et al. reported a non-statistically significant difference in PIA occurrence between groups in their study, which became a statistically significant effect in our meta-analysis [OR 0.46 (0.30–0.70)]. They performed a parallel randomized clinical trial on 223 patients, with approximately 10 sessions per patient (1,843 sessions in total). In their analysis, they corrected for serial comparisons with a *post-hoc* Bonferroni correction. We bundled the individual patient data and corrected it with the ICC; thus, no *post-hoc* analysis was needed. In contrast, three other included studies (17, 18, 29) reported statistically significant differences, which became non-statistically significant effect sizes in our meta-analysis. This was caused by the dichotomization of the outcome and the ICC correction.

Furthermore, in the analysis of the cross-over trials, we lost data on the studies that included different dexmedetomidine doses as interventions (14, 27) due to statistical constraints. Lastly, meta-regression was considered to investigate a dose–response relation of dexmedetomidine but ultimately we decided against this. The main reason was the apprehension of overanalyzing the data resulting in a conclusion that might be speculative.

### 4.4. Clinical applicability of dexmedetomidine

Dexmedetomidine has sedative, anxiolytic, analgesic, and sympatholytic properties as a highly selective alpha-2 agonist. It is an established drug in procedural sedation and for the treatment of (postoperative) delirium and other cognitive disturbances in the intensive care unit (37). The clinical applicability of dexmedetomidine in ECT is not hampered with regard to safety, the impact on seizure characteristics, or administration. The contraindications for dexmedetomidine are relatively sparse (i.e., bradyarrhythmias, clinically significant heart blocks, and significant cardiac valvular stenosis), and Li et al. (12) found that dexmedetomidine does not significantly alter seizure duration or recovery time. Lastly, the administration is intravenous, as is the regular anesthesia. Of note, a sublingual formulation has recently been developed and tested in phase III studies for acute agitation in patients with bipolar disorder, schizophrenia, and schizoaffective disorder (38, 39). On the other hand, the costs for intravenous administration of dexmedetomidine are relatively high (approximately 23 euro/dose), especially when compared to the similar drug clonidine (approximately 1.5 euro/dose). Currently, further research into the preventative effect of clonidine in postictal delirium after ECT (NCT04828226) is being done. Overall, the safety and applicability of dexmedetomidine seem favorable for wider use in the ECT setting.

### 4.5. Conclusion

This review summarized the current evidence for (preventative) pharmacological interventions in PIA management after ECT. For clinical practice, we believe that the results indicate that dexmedetomidine should be considered for the prevention of PIA in patients that have experienced PIA before. The low heterogeneity in the meta-analysis and convincing effect size support this recommendation and outweigh the relatively high risk of bias in the individual studies. The high recurrence rate of PIA (19–54%) and serious adverse effects for the patient and staff further support the need for a preventative approach.

## Data availability statement

The original contributions presented in the study are included in the article/Supplementary material, further inquiries can be directed to the corresponding author.

## Author contributions

TF, DR, and AB conceptualized the systematic review and meta-analysis. TF, YB, and KK performed the literature search. TF and YB performed the data extraction. AH and TF performed the statistical analyses. TF, YB, and AH wrote the first draft and revised the versions of the manuscript. All authors revised the manuscript critically and approved the final manuscript.
