# Supplementary material for: Pharmacological prevention of postictal agitation after electroconvulsive therapy—A systematic review and meta-analysis
Source: Front Psychiatry. 2023 Apr 20;14:1170931. doi: 10.3389/fpsyt.2023.1170931 (PMC10157235; doi:10.3389/fpsyt.2023.1170931)
Supplement: Supplementary file 1 [file Data_Sheet_1.docx]

Supplementary Material

Pharmacological prevention of postictal agitation after electroconvulsive therapy – a systematic review and meta-analysis.

Thomas C Feenstra^1,2^ , Yvonne Blake^1^, Adriaan W Hoogendoorn^2,4^, Krista Koekenbier^3,5^, Aartjan TF Beekman^2,3,4^, Didi Rhebergen^1,2,4^

^1^GGZ Centraal Mental Health Care, Amersfoort, The Netherlands

^2^Amsterdam Public Health Research Institute, Mental Health Program, Amsterdam, The Netherlands

^3^GGZ inGeest Mental Health Care, Amsterdam, The Netherlands

^4^Amsterdam UMC location Vrije Universiteit Amsterdam, Department of Psychiatry, Amsterdam, The Netherlands

^5^Leiden University Medical Center, Department of Psychiatry, Leiden, The Netherlands

*** Correspondence:** Thomas C Feenstra, MD: t.feenstra@ggzcentraal.nl

# Supplementary Tables

## Supplementary Table 1: Search string for systematic review

**Supplement Table 2:** Search string for systematic review

| Databases | Search Terms |
| --- | --- |
| Pubmed | - #ECT "Electroconvulsive Therapy"[Mesh] OR electroconvulsive therap*[tiab] OR electroconvulsive shock*[tiab] OR electroconvulsive treatment*[tiab] OR electroshock*[tiab] OR ECT[tiab] OR electric shock therap*[tiab] OR electric convulsive therap*[tiab] - # Postictal confusion "Confusion"[Mesh] OR "Psychomotor Agitation"[Mesh] OR post-ictal delir* [tiab] OR postictal delir* [tiab] OR interictal delir* [tiab] OR emergence delir* [tiab] OR emergence agitat* [tiab] OR confus* [tiab] OR disorient* [tiab] OR (( agitat* [tiab] OR excitement [tiab] OR restlessness [tiab] OR delir* [tiab]) AND (post-ictal [tiab] OR postictal [tiab] OR inter-ictal [tiab] OR interictal [tiab] OR post-ect [tiab] OR postconvulsive [tiab] OR ECT-induced [tiab] OR during* [tiab])) - # Hyperd* |
| PsychINFO (EBSCO) | - #ECT DE "Electroconvulsive Shock" OR DE "Electroconvulsive Shock Therapy" OR TI(“electroconvulsive shock*” OR “electroconvulsive treatment*” OR electroshock* OR ECT OR “electric shock therap*” OR “electric convulsive therap*”) OR AB(“electroconvulsive shock*” OR “electroconvulsive treatment*” OR electroshock* OR ECT OR “electric shock therap*” OR “electric convulsive therap*”) OR KW(“electroconvulsive shock*” OR “electroconvulsive treatment*” OR electroshock* OR ECT OR “electric shock therap*” OR “electric convulsive therap*”) - #Postictal confusion DE "Mental Confusion" OR DE "Restlessness" OR DE "Agitation" OR TI(“emergence delir*”OR “emergence agitat*” OR confus* OR disorient* OR (( agitat* OR excitement OR restlessness OR delir*) AND (“post-ictal” OR postictal OR “inter-ictal” OR interictal OR “post-ect” OR postconvulsive OR “ECT-induced” OR during*)) OR AB(“emergence delir*”OR “emergence agitat*” OR confus* OR disorient* OR (( agitat* OR excitement OR restlessness OR delir*) AND (“post-ictal” OR postictal OR “inter-ictal” OR interictal OR “post-ect” OR postconvulsive OR “ECT-induced” OR during*)) OR KW(“emergence delir*”OR “emergence agitat*” OR confus* OR disorient* OR (( agitat* OR excitement OR restlessness OR delir*) AND (“post-ictal” OR postictal OR “inter-ictal” OR interictal OR “post-ect” OR postconvulsive OR “ECT-induced” OR during*)) - #hyperdynamic - DE "hyperd*" OR TI "hyperd*" OR AB "hyperd*" OR "hyperd*" OR DE "hyperdynamic" OR TI "hyperdynamic" OR AB "hyperdynamic" OR KW "hyperdynamic" OR "hyperdynamic" - Filters: (Limiters: academic journals) |
| Embase (Embase.com) | - #ECT 'electroconvulsive therapy'/exp OR 'electroconvulsive therap*’:ab,ti,kw OR 'electroconvulsive shock*’:ab,ti,kw OR 'electroconvulsive treatment*’:ab,ti,kw OR electroshock*:ab,ti,kw OR ECT:ab,ti,kw OR 'electric shock therap*’:ab,ti,kw OR 'electric convulsive therap*’:ab,ti,kw - #Postictal confusion 'confusion'/exp OR 'restlessness'/exp OR 'agitation'/exp OR 'emergence agitation'/exp OR 'emergence delir*’:ab,ti,kw OR 'emergence agitat*’:ab,ti,kw OR confus*:ab,ti,kw OR disorient*:ab,ti,kw OR ((agitat* OR excitement OR restlessness OR delir*) NEAR/3 (post-ictal OR postictal OR inter-ictal OR interictal OR post-ect OR postconvulsive OR ECT-induced OR during*)):ab,ti,kw - #hyperdynamic - ‘hyperdynamic circulation’:ab,ti,kw OR ‘hyperd*:ab,ti,kw, - Filters: ([article]/lim OR [article in press]/lim OR [editorial]/lim OR [letter]/lim OR [review]/lim OR [short survey]/lim) |
| Web of Science (Clarivate) | - TS=(“electroconvulsive shock*” OR “electroconvulsive treatment*” OR electroshock* OR ECT OR “electric shock therap*” OR “electric convulsive therap*”) - TS=(“emergence delir*” OR “emergence agitat*” OR confus* OR disorient*) - TS=((agitat* OR excitement OR restlessness OR delir*) AND (“post-ictal” OR postictal OR “inter-ictal” OR interictal OR “post-ect” OR postconvulsive OR “ECT-induced” OR during*)) - TI=(“hyper*” OR “hyperdynamic”) - Filters: Indexes=SCI-EXPANDED, SSCI, A&HCI, ESCI Timespan=All years |
|  | |

## Supplementary Table 2: List of included studies and patient characteristics

**Supplementary Table 2:** List of included studies and patient characteristics

| **Study ID** | **Population** |  |  |  | **Treatment** |  | **Patient characteristics** | | **Outcome** |  |
| --- | --- | --- | --- | --- | --- | --- | --- | --- | --- | --- |
| *Author (year), country,*  *study design* | *Inclusion- and exclusion criteria^*^* | *Sample size, no. ECT sessions* | *ECT technique* | *General anesthesic procedure* | *Intervention(s)* | *Comparison^**^* | *Mean age (SD or range)* | *Female (%)* | *Outcome scale* | *Effect^***^, p-value if reported* |
| **Cross-over trials on dexmedetomidine** | | | | | | | | | | |
| Moshiri (25) (2016), Iran  Double-blind, randomized cross-over trial | Patients, 18-50 years, ASA II, indicated for ECT.  Exclusion if: >60% energy was needed by ECT, pregnancy, cardiovascular disease, β-blockers or narcotics use, hemodynamic instability, seizure < 25s | N=25  75 sessions | Bitemporal stimulation, 30-60% energy | *Premedication:*  atropine  *Anesthesia:*  thiopental 2-3mg/kg  *Muscle relaxant:*  succinylcholine 0.5mg/kg | 1. dexmedetomidine 0.5mcg/kg  2. alfentanil 10mcg/kg | saline | Males: 37.5 years (10.8)  Females: 39.6 years (12.5) | 60% | Emergence agitation score (1-5)  (1= sleeping, 2= awake and calm, 3= irritable and crying, 4= inconsolable crying, 5= severe restlessness and disorientation  purposelessly wanting to get out of the bed, wanting to  stand on the bed, shouting, crying, or mumbling loudly)  PIA defined as a score ≥ 3 | The mean agitation score in the alfentanil group was 1.84 (0.37), in dexmedetomidine group 1.96 (0.2) and 2.06 (0.04) in the placebo group over 25 sessions. No statistically significant difference was observed between these groups. |
| Parikh (26) (2017), India  Double-blind, randomized cross-over trial | Patients, 18-50 years, ASA I, scheduled for ≥ 4 ECT sessions  Exclusion if: pregnancy of lactating females | N=30  150 sessions | Bitemporal stimulation, determined by ECT treatments received before entering the study. | *Premedication:* glycopyrrolate 0.2mg  *Anesthesia:*  propofol 1mg/kg  *Muscle relaxant:*  succinylcholine 0.5mg/kg | 1. dexmedetomidine 0.5mcg/kg  2. dexmedetomidine 1mcg/kg  3. esmolol 1mg/kg  4. lignocaine 1mg/kg | saline | 6/30 patients were 40-50 years old and 24/30 were 18-40 years old. | 57% | Emergence agitation score (1-5)  PIA defined as a score ≥ 3 | The mean agitation score showed a statistically significant difference between the comparison (1.83±0.74) and intervention group 2 (1.33±0.471) with a  p-value of 0.006. The mean agitation scores showed no statistically significant difference between the comparison and intervention group 1,3 or 4.  Furthermore they reported:  Intervention 1: 0/30 sessions with postictal agitation  Intervention 2: 0/30 sessions with postictal agitation  Intervention 3: 0/30 sessions with postictal agitation  Intervention 4: 0/30 sessions with postictal agitation  Comparison: 0/30 sessions with postictal agitation |
| Mizrak (17) (2009), Turkey  Double-blind, randomized cross-over trial | Patients, over 18 years old, ASA I or II, indicated for ECT, whom had severe post-ECT agitation more than once before.  Exclusion if: pregnancy, ischemic heart disease, heart blocks, arrhythmia, β-adrenergic blockers, tricyclic antidepressants usage, and family history of adverse reactions to dexmedetomidine  or midazolam | N=15  45 sessions | Bitemporal stimulation, 1.5x seizure threshold, Funes Electronic, Gaziantep, Turkey | *Premedication:* none  *Anesthesia:*  propofol 20mg/10s, dose-titration method  *Muscle relaxant:*  succinylcholine 0.5mg/kg | 1. dexmedetomidine 0.5mcg/kg  2. midazolam 0.025mg/kg  (pre-ECT) | saline | 32 years (9) | 33% | Emergence agitation score (1-5)  PIA defined as a score ≥ 3 | The agitation scores in groups 1 or 2 were significantly lower than those in the control group at 10 and 15 min after electroconvulsive therapy (ECT) with an associated p-value < 0.05.  Furthermore they reported:  Intervention 1: 0/15 sessions with agitation score 4-5  Intervention 2: 0/15 sessions with agitation score 4-5  Comparison: 3/15 sessions with agitation score 4-5  The number of sessions with a score of 3 was unextractable. No statistical test was performed on these differences |
| Subsoontorn (14) (2021), Thailand  Double-blind, randomized cross-over trial | Patients, 18-70 years old, ASA I to III, indicated for ECT by the psychiatrist  Exclusion if: liver disease, severe ventricular dysfunction, advanced heart block, pregnancy, β-blockers or narcotics use. | N=24  72 sessions | Not noted | *Premedication:* none  *Anesthesia:*  thiopental 2-3mg/kg or propofol 1-2mg/kg  *Muscle relaxant:* succinylcholine 1-2 mg/kg | 1.dexmedetomidine 0,5mcg/kg  2.dexmedetomidine 1mcg/kg | saline | 44.83 years (12.71) | 58,3% | Postictal agitation  4-point rating scale  (1= calm or asleep, 2= restless but calmed down when talked to, 3= restless and required a nurse to stand next to the bed, 4= one or more nurses were required to physically hold down the patient)  PIA defined as a score ≥ 3 | Intervention 1: 0/24 sessions with postictal agitation  Intervention 2: 0/24 sessions with postictal agitation  Comparison: 3/24 sessions with postictal agitation  Chi-square test or fisher’s exact test, p-value: 0.052 |
| Sannakki (27) (2017), India  Double-blind, randomized cross-over trial | Patients, 18-50 years old, ASA I to II, indicated for ECT by the psychiatrist  Exclusion: involuntary patient status, pregnancy, lactation, asthma, drugs such as beta blockers, calcium channel blockers, digitalis, history of myocardial infarction, congestive cardiac failure in previous 6 months, significant arrhythmias, known family history of reactions to study drugs. | N=30  60 sessions | Bifrontotemporal stimulation | *Premedication:* none  *Anesthesia:*  propofol 1mg/kg  *Muscle relaxant:*  succinylcholine 0.5mg/kg | dexmedetomidine 1mcg/kg | saline | 31.37 years (9.60) | 37% | Richmond Agitation-Sedation Scale (RASS)  PIA defined as a RASS ≥ 2 | Intervention: 0/30 sessions with postictal agitation  Comparison: 0/30 sessions with postictal agitation |
| **Parallel RCT on dexmedetomidine** | | | | | | | | | | |
| Qiu (13) (2020), China  Double-blind, parallel randomized controlled trial | Patients, 16-55 years old, ASA I or II, indicated for ECT for a psychiatric condition  Exclusion if: severe hepatic or renal insufficiency, organic brain disease, cardiac insufficiency, sick sinus syndrome, bradycardia, atrioventricular block  (degrees II and III). | N=223  1843 sessions | Bitemporal stimulation, ‘half-age’ stimulation strategy, brief pulse (0.5ms), Thymatron IV System (Somatics, Lake Bluf, Illinois,USA) | *Premedication:*  atropine 0.006 mg/kg  *Anesthesia:*  propofol 1.5mg/kg  *Muscle relaxant:* succinylcholine 0.7 mg/kg | dexmedetomidine 0.5mcg/kg | saline | Intervention  25.3 years (11.2)  Comparison  25.5 years (10.8) | Intervention  59%  Comparison  61% | Richmond Agitation-Sedation Scale  PIA defined as RASS ≥ 2 | Intervention (111 patients): 92/909 sessions with postictal agitation  Comparison (112 patients): 183/934 sessions with postictal agitation  Chi-square test followed by post-hoc Bonferroni correction which showed no statically significant difference between groups, p-value not reported. |
| Shams (28) (2014), Egypt  Double-blind, parallel randomized controlled trial | Patients, 18-60 years old, ASA I or II with a depression indicated for ECT.  Exclusion if: serious physical disease, such as cardio- or cerebrovascular disease, intracranial hypertension,  respiratory tract disease, or a previous fracture, glaucoma, arterial aneurysm, cerebrovascular malformation, pacemaker, history of seizures & pregnancy. | N=40  280 sessions | Bifrontotemporal stimulation | *Premedication:*  atropine 0.5 mg  *Anesthesia:*  10 mg/ml ketamine and 10 mg/ml propofol mixed in a 20 ml syringe, dose titration method  *Muscle relaxant:*  succinylcholine 0.5 mg/kg | dexmedetomidine 0.5mcg/kg | saline | Intervention  36.5 years (10.2)  Comparison  34.9 years (9.7) | Intervention  30%  Comparison  25% | Emergence agitation score (1-5)  PIA defined as a score ≥ 3 | Intervention (20 patients): 2/140 sessions with postictal agitation  Comparison (20 patients): 12/140 sessions with postictal agitation  Chi-square test or fisher’s exact test, p-value: 0.014 |
| Li (29) (2017), China  Double-blind, parallel randomized controlled trial | Patients, 16-55 years old, ASA I or II, indicated for ECT for a psychiatric condition  Exclusion if: severe hepatic or renal insufficiency, organic brain disease, cardiac insufficiency, sick sinus syndrome,  bradycardia, atrioventricular block of degree II and III | N=78  468 sessions | Bitemporal stimulation, dose-titration method, Thymatron System IV | *Premedication:*  Atropine 0.006 mg/kg  *Anesthesia:* 1.5mg/kg propofol  *Muscle relaxant:* 0.7 mg/kg succinylcholine | dexmedetomidine 0.2mcg/kg | saline | Intervention  29.97 years (10.56)  Comparison  28.28 years (11.09) | Intervention  46%  Comparison  36% | Emergence agitation score (1-5)  PIA defined as a score ≥ 3 | Intervention (39 patients): 36/234 sessions with postictal agitation  Comparison (39 patients): 58/234 sessions with postictal agitation  Chi-square test or fisher’s exact test, p-value: 0.011 |
| Bagle (30) (2016), India  Double-blind, parallel randomized controlled trial | Patients, 18-60 years old, ASA I or II, indicated for ECT  Exclusion if: serious physical disease, such as cardiovascular disease, cerebrovascular disorder, intracranial hypertension, respiratory  tract disease, previous fracture, glaucoma, arterial aneurysm,  history of seizures, status, baseline bradycardia, use of beta-blockers or narcotics and pregnancy. | N=60  60 sessions | Bifrontotemporal stimulation, pulse width 0.1s, constant-current  ECT device with a current of 120 mC and frequency of 70 Hz | *Premedication:*  ondansetron 4mg and glycopyrrolate  0.2mg  *Anesthesia:*  thiopentone 3mg/kg  *Muscle relaxant:*  Succinylcholine 0.5 mg/kg | dexmedetomidine 0.5mcg/kg | saline | Intervention  35.7 years (7.97)  Comparison  37.47 years (8.54) | Intervention  43%  Comparison  53% | Emergence agitation score (1-5)  PIA defined as a score ≥ 3 | Intervention (30 patients): 0/30 sessions with postictal agitation  Comparison (30 patients): 3/30 sessions with postictal agitation  Chi-square test, p-value: 0.236 |
| Kumar (31) (2020), Iraq  Double-blind, parallel randomized controlled trial | Patients, 18-60 years old, ASA I or II, with major depressive disorder indicated for ECT.  Exclusion if: baseline bradycardia, other psychiatric disorders, serious physical disease (cardio or cerebrovascular disease, ICH, respiratory tract disease, glaucoma, previous fracture, history of seizures, hemodynamic instability), pregnancy | N=92  92 sessions | Bifrontotemporal stimulation | *Premedication:*  atropine 0.5 mg  *Anesthesia:*  ketamine 10 mg/ml and 10mg/ml propofol, dose titration method  *Muscle relaxant:*  succinylcholine 0.5mg/kg | dexmedetomidine 0.5mcg/kg | saline | Intervention  44.95 years (11.041)  Comparison  43.08 years (12.98) | Intervention  78.26%  Comparison  28.26% | Agitation scale consisting of 3 points.  (1= sleeping,  2= awake and calm, 3= irritable and crying)  PIA defined as a score of 3 | The mean agitation score showed a statistically significant difference between the comparison (1.27 ± 0.577) and intervention group (1.04 ± 1.203) with a  p-value of 0.0125.  Furthermore they reported:  Intervention (46 patients): 0/46 sessions with postictal agitation  Comparison (46 patients): 3/46 sessions with postictal agitation  No statistical test was performed on these differences |
| **Studies on other drugs** | | | | | | | | | | |
| Gaddam (15) (2022), India  Double-blind, cross-over trial | Patients, 18-65 years, ASA I or II, indicated for ECT  Exclusion if: cardiovascular diseases, chronic opiate use, pregnancy, lactation | N=30  90 sessions | Bifrontotemporal stimulation | *Premedication:*  glycopyrrolate 0.004mg/kg  *Anesthesia:*  see intervention  *Muscle relaxant:*  succinylcholine 0.75 mg/kg | 1. ketofol (propofol 0.5mg/kg and ketamine 0.5mg/kg)  2. propofol 1mg/kg  3. thiopentone 3mg/kg | none | 31.63 years ± 10.19 | 57% | Emergence agitation score (1-5)  PIA defined as a score ≥ 3 | Mean agitation score was highest in intervention group 3 (2.13 ± 0.57), showed a statistically significant difference when compared to intervention group 2 (1.63 ± 0.49) and group 1 (1.77 ± 0.63) with a p-value of 0.003.  Furthermore they reported:  Intervention 1: 3/30 sessions with postictal agitation  Intervention 2: 0/30 sessions with postictal agitation  Intervention 3: 7/30 sessions with postictal agitation  No statistical test was performed on these differences |
| Tzabazis (32) (2013), Germany  Patient blinded, cross-over trial | Patients, indicated for ECT for severe depression not responding to pharmacological interventions  Exclusion not specified in article, 1 exclusion due to supraventricular tachycardia. | N=13  74 ECT sessions | Unilateral stimulation, brief pulse, Thymatron System II | *Premedication:*  none  *Anesthesia:*  etomidate 0.15-0.2 mg/kg  *Muscle relaxant:* succinylcholine 0.9-1.2 mg/kg | propofol 0.5mg/kg (post-ECT) | none | 46 years (23-61)  . | 54% | 4-point numeric rating scale  (1= no agitation, 2= mild agitation; 3= moderate agitation 4= severe agitation)  PIA defined as a score ≥ 1 | Intervention: 3/37 sessions with moderate to severe postictal agitation.  Comparison: 8/37 sessions with moderate to severe postictal agitation  The number of sessions with a score of 1 was not extractable with certainty.  Generalized linear model, p-value: <0.05 |
| Rasmussen (33) (2013), USA  Double-blind, parallel randomized controlled trial | presence of non-psychotic major depressive episode, whether unipolar or bipolar and indicated for ECT.  Exclusion if: psychotic of major neurological disorder | N=35  166 sessions | Bitemporal stimulation, 1.5x seizure threshold, brief pulse (1.0ms) or unilateral stimulus at 6x seizure threshold, ultrabrief pulse (0.25ms), Thymatron DGX | *Premedication:*  glycopyrrolate not further specified  *Anesthesia:*  see intervention  *Muscle relaxant:*  succinylcholine not further specified | ketamine 1.0mg/kg | methohexital 1.0mg/kg | Intervention  47.0 years (13.2)  Comparison  48.6 years (7.2) | Intervention  76%  Comparison  47% | 4-point PIA rating scale  (0= absent, 1= mild, 2= moderate, 3= severe) | Intervention (mean score) (84 patients): 0.07 (0.26) over 84 sessions  Comparison (mean score) (82 patients): 0.09 (0.36) over 82 sessions  Repeated measures longitudinal models, p-value: 0.860 |
| Gomez (34) (1975), England  Partly randomized (due to pilot phase), single-blind (rater), cross-over trial | Patients with severe depression or with a depression who fail to respond satisfactorily to antidepressants, whom are indicated for ECT.  Exclusion if: too  disturbed or too sedated to complete a simple rating scale before starting ECT. | N=40  120 sessions | Unilateral stimulation in 30 patients, bilateral stimulation in 10 patients. | *Premedication:*  atropine not further specified  *Anesthesia:*  thiopentone not further specified  *Muscle relaxant:*  succinylcholine not further specified | 1. intravenous haloperidol 20mg (and procyclidine 10mg to lessen dystonic side-effects) (pre-ECT)  2. intravenous diazepam 20mg (pre-ECT) | none | 51 years (34-79) | 73% | 4-point agitation scale  (0= calm and composed, 1 = talkative and unable to relax, 2= restless and pressure to talk, 3= very restless and noisy) | The mean agitation score showed a statistically significant difference between the comparison (1.25) and intervention group 1 (0.27) and intervention group 2 (0.36) with a p-value < 0.01. |

*Only study specific exclusion criteria were mentioned in this table. Evident exclusion criteria that match international ECT guidelines (i.e. recent stroke, intracranial surgery, feochromocytoma, unstable coronary heart disease, allergy or contra-indication to study drug) were not specified in this table.

** When saline was used as a comparison, all studies used similar quantities of saline to assure blinding.

***If the amount of PIA cases is not specified in this column, there was no possibility for the authors to extract these results.

## Supplementary Table 3: GRADE appraisal for the results

**Supplementary Table:** GRADE appraisal for the results

**Question:** Dexmedetomidine compared to placebo for prevention of postictal agitation in patients receiving electroconvulsive therapy

| **Certainty assessment** | | | | | | | **№ of sessions^c^** | | **Effect** | | **Certainty** | **Importance** |
| --- | --- | --- | --- | --- | --- | --- | --- | --- | --- | --- | --- | --- |
| **№ of studies** | **Study design** | **Risk of bias** | **Inconsistency** | **Indirectness** | **Imprecision** | **Other considerations** | **dexmedetomidine** | **placebo** | **Relative (95% CI)** | **Absolute (95% CI)** |  |  |
| 9^a^ | randomised trials | serious^b^ | not serious | not serious | not serious | none | 130/1512 (8.6%) | 265/1483 (17.9%) | **OR 0.45^d^** (0.32 to 0.63) | **90 fewer per 1.000** (from 114 fewer to 58 fewer) | ⨁⨁⨁◯ Moderate | IMPORTANT |

**CI:** confidence interval; **OR:** odds ratio

Explanations

a. Moshiri et al 2016 was not included in the meta-analysis, as the reported comparison mean agitation scores did not reflect postictal agitation in the groups and the dichotomous data on PIA could not be extracted.

b. We downgraded by 1 level, because the risk of bias assessment for all resulted in some concerns of high risk of bias. As the two main contributing studies were rated as some concerns, we decided to downgrade by one level instead of 2 levels.

c. We reported the amount of sessions with PIA here, as it was not exactly known how many individual patients experienced PIA in the sample

d. This OR was calculated through the methods described in the manuscript, not with the no. of sessions reported in this table.

**Question:** Alfentanil 10mcg/kg compared to placebo for prevention in patients receiving electroconvulsive therapy

| **Certainty assessment** | | | | | | | **Impact** | **Certainty** | **Importance** |
| --- | --- | --- | --- | --- | --- | --- | --- | --- | --- |
| **№ of studies** | **Study design** | **Risk of bias** | **Inconsistency** | **Indirectness** | **Imprecision** | **Other considerations** |  |  |  |
| 1 | randomised trials | very serious^a^ | not serious | very serious^b^ | not serious | none | This study used the emergence agitation scale (1 = sleepy, 2 = awake and peaceful, 3 = irritable and noisy, 4 = disconsolate noisy, and 5 = severe blenched or willing to wake up from bed or sitting on the bed and shrieking). The mean agitation score in the alfentanil group was 1.84 (0.37) over 25 sessions and 2.06 (0.04) in the placebo group over 25 sessions. No statistically significant difference was observed between these groups. | ⨁◯◯◯ Very low | NOT IMPORTANT |

Explanations

a. We downgraded by 2 levels, because the risk of bias assessment showed high risk of bias.

b. We downgraded by 2 levels for severe indirectness, as mean agitation scores do not reflect postictal agitation in groups. Therefore, we believe that this analysis is not suitable to measure postictal agitation differences between groups.

**Question:** Lignocaine 1mg/kg compared to placebo for prevention of postictal agitation in patients receiving electroconvulsive therapy

| **Certainty assessment** | | | | | | | **Impact** | **Certainty** | **Importance** |
| --- | --- | --- | --- | --- | --- | --- | --- | --- | --- |
| **№ of studies** | **Study design** | **Risk of bias** | **Inconsistency** | **Indirectness** | **Imprecision** | **Other considerations** |  |  |  |
| 1 | randomised trials | serious^a^ | not serious | very serious^b^ | not serious | none | This study used the emergence agitation scale (1 = sleepy, 2 = awake and peaceful, 3 = irritable and noisy, 4 = disconsolate noisy, and 5 = severe blenched or willing to wake up from bed or sitting on the bed and shrieking). The mean agitation score in the lignocaine group was 1.6 (0.53) over 30 sessions and 1.83 (0.74) in the placebo group over 30 sessions. No statistically significant difference was observed between these groups. | ⨁◯◯◯ Very low | NOT IMPORTANT |

Explanations

a. We downgraded by 1 level, because the risk of bias assessment revealed an overall unclear risk of bias.

b. We downgraded by 2 levels for severe indirectness, as mean agitation scores do not reflect postictal agitation in groups. Therefore, we believe that this analysis is not suitable to measure postictal agitation differences between groups.

**Question:** Esmolol 1mg/kg compared to placebo for prevention of postictal agitation in patients receiving electroconvulsive therapy

| **Certainty assessment** | | | | | | | **Impact** | **Certainty** | **Importance** |
| --- | --- | --- | --- | --- | --- | --- | --- | --- | --- |
| **№ of studies** | **Study design** | **Risk of bias** | **Inconsistency** | **Indirectness** | **Imprecision** | **Other considerations** |  |  |  |
| 1 | randomised trials | serious^a^ | not serious | very serious^b^ | not serious | none | This study used the emergence agitation scale (1 = sleepy, 2 = awake and peaceful, 3 = irritable and noisy, 4 = disconsolate noisy, and 5 = severe blenched or willing to wake up from bed or sitting on the bed and shrieking). The mean agitation score in the esmolol group was 1.9 (0.66) over 30 sessions and 1.83 (0.74) in the placebo group over 30 sessions. No statistically significant difference was observed between these groups. None of the patients had emergence agitation. | ⨁◯◯◯ Very low | NOT IMPORTANT |

Explanations

a. We downgraded by 1 level, because the risk of bias assessment revealed an overall unclear risk of bias.

b. We downgraded by 2 levels for severe indirectness, as mean agitation scores do not reflect postictal agitation in groups. Therefore, we believe that this analysis is not suitable to measure postictal agitation differences between groups.

**Question:** Midazolam 0.025mg/kg (pre-ECT) compared to placebo for prevention of postictal agitation in patients receiving electroconvulsive therapy

| **Certainty assessment** | | | | | | | **Impact** | **Certainty** | **Importance** |
| --- | --- | --- | --- | --- | --- | --- | --- | --- | --- |
| **№ of studies** | **Study design** | **Risk of bias** | **Inconsistency** | **Indirectness** | **Imprecision** | **Other considerations** |  |  |  |
| 1 | randomised trials | serious^a^ | not serious | serious^b^ | not serious | none | This study used the emergence agitation scale (1 = sleepy, 2 = awake and peaceful, 3 = irritable and noisy, 4 = disconsolate noisy, and 5 = severe blenched or willing to wake up from bed or sitting on the bed and shrieking). They reported significantly lower mean agitation scores at 10 and 15 minutes after ECT for midazolam in comparison to control. The results were unextractable, because they were reported in a figure only. Furthermore, they reported that 3 patients in the control group needed additional medication for agitation scores of 4 and 5, as opposed to 0 in the intervention group. | ⨁⨁◯◯ Low | NOT IMPORTANT |

Explanations

a. We downgraded by 1 level, because the risk of bias assessment revealed an overall unclear risk of bias.

b. We downgraded by 1 level for severe indirectness, as mean agitation scores do not reflect postictal agitation in groups. Therefore, we believe that this analysis is not suitable to measure postictal agitation differences between groups. However, as the dichotomized data supported a difference in PIA cases (3 in the comparison as opposed to 0 in the intervention group), we did not downgrade by 2 levels.

**Question:** Ketofol (propofol 0.5mg/kg and ketamine 0.5mg/kg) compared to thiopentone 3mg/kg in anesthesia for prevention of postictal agitation in patients receiving electroconvulsive therapy

| **Certainty assessment** | | | | | | | **№ of patients** | | **Effect** | | **Certainty** | **Importance** |
| --- | --- | --- | --- | --- | --- | --- | --- | --- | --- | --- | --- | --- |
| **№ of studies** | **Study design** | **Risk of bias** | **Inconsistency** | **Indirectness** | **Imprecision** | **Other considerations** | **ketofol (propofol 0.5mg/kg and ketamine 0.5mg/kg)** | **thiopentone 3mg/kg** | **Relative (95% CI)** | **Absolute (95% CI)** |  |  |
| 1 | randomised trials | very serious^a^ | not serious | not serious | not serious | none | 3/30 (10.0%) | 7/30 (23.3%) | not estimable | not estimable | ⨁⨁◯◯ Low | NOT IMPORTANT |

Explanations

a. We downgraded by 2 levels as risk of bias assessment revealed an overall high risk.

**Question:** Propofol 1mg/kg compared to thiopentone 3mg/kg in anesthesia for prevention of postictal agitation in patients receiving electroconvulsive therapy

| **Certainty assessment** | | | | | | | **№ of patients** | | **Effect** | | **Certainty** | **Importance** |
| --- | --- | --- | --- | --- | --- | --- | --- | --- | --- | --- | --- | --- |
| **№ of studies** | **Study design** | **Risk of bias** | **Inconsistency** | **Indirectness** | **Imprecision** | **Other considerations** | **propofol 1mg/kg** | **thiopentone 3mg/kg** | **Relative (95% CI)** | **Absolute (95% CI)** |  |  |
| 1 | randomised trials | very serious^a^ | not serious | not serious | not serious | none | 0/30 (0.0%) | 7/30 (23.3%) | not estimable | not estimable | ⨁⨁◯◯ Low | NOT IMPORTANT |

Explanations

a. We downgraded by 2 levels as the risk of bias assessment revealed an overall high risk.

**Question:** Propofol 0.5mg/kg (post-ECT) compared to placebo for prevention of postictal agitation in patients receiving electroconvulsive therapy

| **Certainty assessment** | | | | | | | **№ of patients** | | **Effect** | | **Certainty** | **Importance** |
| --- | --- | --- | --- | --- | --- | --- | --- | --- | --- | --- | --- | --- |
| **№ of studies** | **Study design** | **Risk of bias** | **Inconsistency** | **Indirectness** | **Imprecision** | **Other considerations** | **propofol 0.5mg/kg (post-ECT)** | **placebo** | **Relative (95% CI)** | **Absolute (95% CI)** |  |  |
| 1 | randomised trials | very serious^a^ | not serious | not serious | not serious | none | 3/37 (8.1%) | 8/37 (21.6%) | not estimable | not estimable | ⨁⨁◯◯ Low | NOT IMPORTANT |

Explanations

a. We downgraded by 2 levels as the risk of bias assessment revealed an overall high risk.

**Question:** Ketamine 1mg/kg compared to methohexital 1mg/kg in anesthesia for prevention of postictal agitation in patients receiving electroconvulsive therapy

| **Certainty assessment** | | | | | | | **Impact** | **Certainty** | **Importance** |
| --- | --- | --- | --- | --- | --- | --- | --- | --- | --- |
| **№ of studies** | **Study design** | **Risk of bias** | **Inconsistency** | **Indirectness** | **Imprecision** | **Other considerations** |  |  |  |
| 1 | randomised trials | very serious^a^ | not serious | not serious | serious^b^ | none | This study used a postictal agitation scale (0=absent, 1=mild, 2=moderate, 3=severe), which was not further specified. The mean agitation score in the ketamine group was 0.07 (0.26) over 84 sessions and 0.09 (0.36) in the methohexital group over 82 sessions. No statistically significant difference was observed between these groups. | ⨁◯◯◯ Very low | NOT IMPORTANT |

Explanations

a. We downgraded by 2 levels, because the risk of bias assessment revealed an overall high risk.

b. We downgraded by 1 levels for imprecision, as mean agitation scores do not reflect postictal agitation in groups. Therefore, we believe that this analysis is not suitable to measure postictal agitation differences between groups.

**Question:** Haloperidol 20mg (pre-ECT and in combination with procyclidine) compared to no medication for prevention of postictal agitation in patients receiving electroconvulsive therapy

| **Certainty assessment** | | | | | | | **Impact** | **Certainty** | **Importance** |
| --- | --- | --- | --- | --- | --- | --- | --- | --- | --- |
| **№ of studies** | **Study design** | **Risk of bias** | **Inconsistency** | **Indirectness** | **Imprecision** | **Other considerations** |  |  |  |
| 1 | randomised trials | very serious^a^ | not serious | very serious^b^ | not serious | none | A 4-point agitation scale was used (0=calm and composed, 1 =talkative and unable to relax, 2=restless and pressure to talk, 3= very restless and noisy). The mean agitation score for haloperidol was 0.27. The control condition had a mean agitation score of 1.25. A statistically significant difference was observed. | ⨁◯◯◯ Very low | NOT IMPORTANT |

Explanations

a. We downgraded by 2 levels, because the risk of bias assessment revealed an overall high risk.

b. We downgraded by 2 levels for severe indirectness, as mean agitation scores do not reflect postictal agitation in groups. Therefore, we believe that this analysis is not suitable to measure postictal agitation differences between groups.

**Question:** Diazepam 20mg (pre-ECT, intravenous administration) compared to no medication for prevention of postictal agitation in patients receiving electroconvulsive therapy

| **Certainty assessment** | | | | | | | **Impact** | **Certainty** | **Importance** |
| --- | --- | --- | --- | --- | --- | --- | --- | --- | --- |
| **№ of studies** | **Study design** | **Risk of bias** | **Inconsistency** | **Indirectness** | **Imprecision** | **Other considerations** |  |  |  |
| 1 | randomised trials | very serious^a^ | not serious | very serious^b^ | not serious | none | A 4-point agitation scale was used (0=calm and composed, 1 =talkative and unable to relax, 2=restless and pressure to talk, 3= very restless and noisy). The mean agitation score for diazepam was 0.36. The control condition had a mean agitation score of 1.25. A statistically significant difference was observed. | ⨁◯◯◯ Very low | NOT IMPORTANT |

Explanations

a. We downgraded by 2 levels, because the risk of bias assessment revealed an overall high risk.

b. We downgraded by 2 levels for severe indirectness, as mean agitation scores do not reflect postictal agitation in groups. Therefore, we believe that this analysis is not suitable to measure postictal agitation differences between groups.

\

# Supplementary Figures

## Supplementary Figure 1: Quality assessment of the included studies

**Supplementary Figure 1:** Quality assessment

**Supplementary Figure 1a:** parallel studies on dexmedetomidine **Supplementary Figure 1b:** cross-over studies on dexmedetomidine.


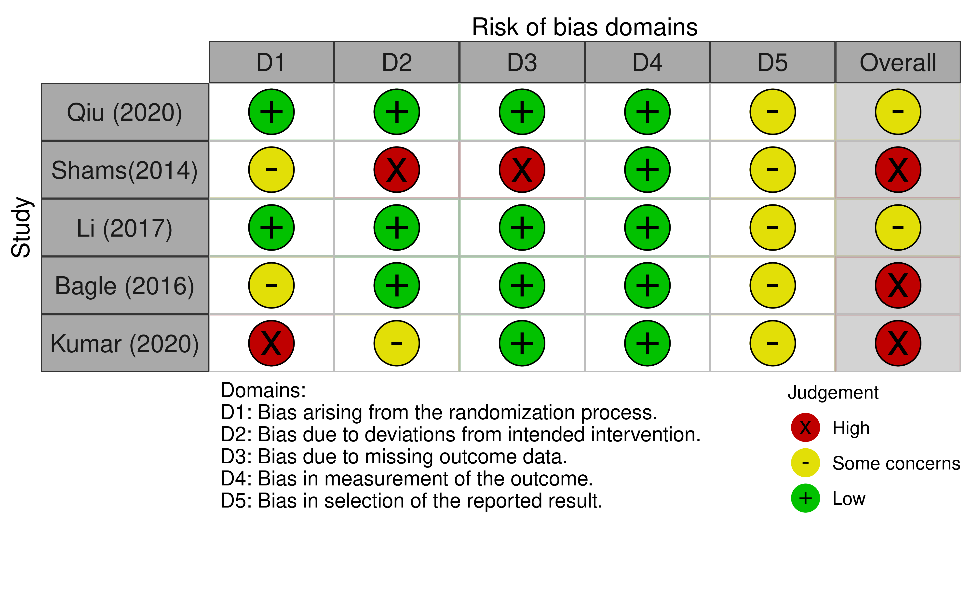

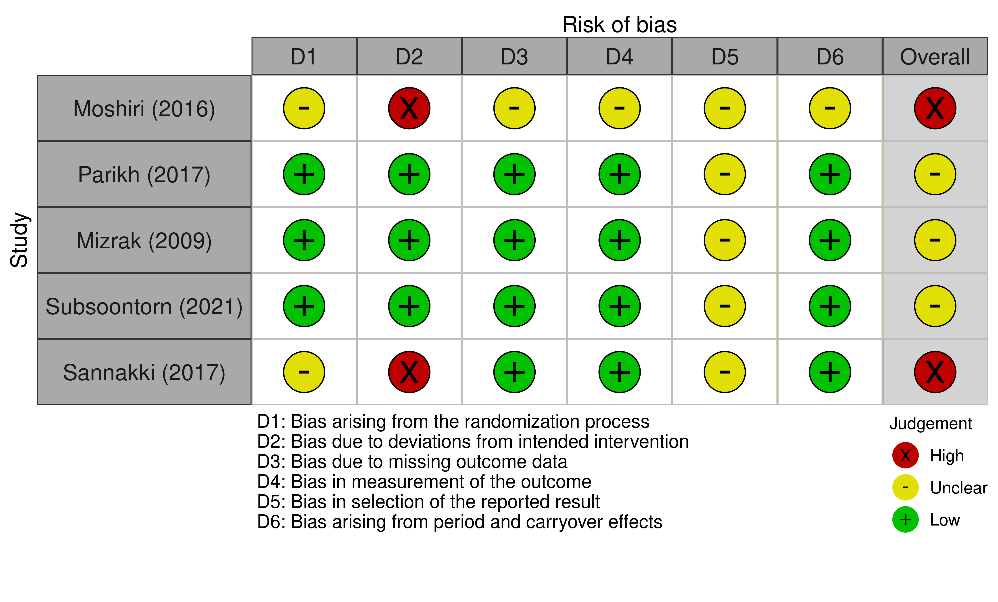


**Supplementary Figure 1c:** cross-over studies on other drugs. **Supplementary Figure 1d:** parallel studies on other drugs.


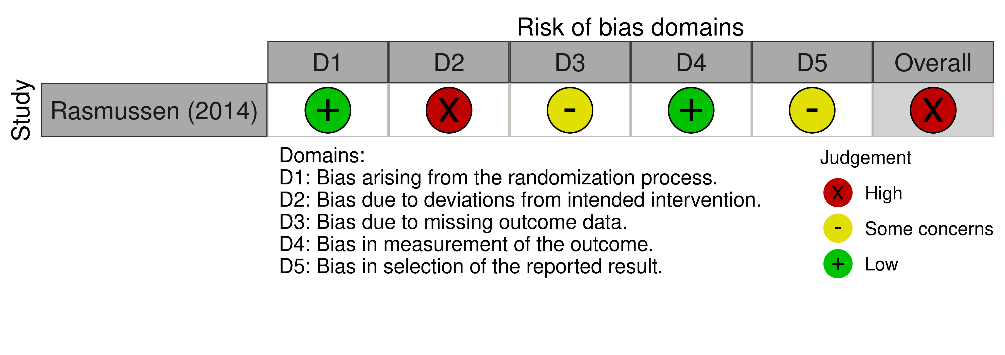


The figures in supplement figure 1 were created using the robvis tool. McGuinness LA, Higgins JPT. Risk-of-bias VISualization (robvis): An R package and Shiny web app for visualizing risk-of-bias assessments. Res Synth Methods. 2021; 12(1): 55-61.
